# Genetic erosion in domesticated barley and a hypothesis of a North African centre of diversity

**DOI:** 10.1002/ece3.70068

**Published:** 2024-08-07

**Authors:** Peter Civáň, Agostino Fricano, Joanne Russell, Caroline Pont, Hakan Özkan, Benjamin Kilian, Terence A. Brown

**Affiliations:** ^1^ INRAE/UCA UMR 1095, GDEC Clermont Ferrand France; ^2^ Council for Agricultural Research and Economics – Research Centre for Genomics and Bioinformatics Fiorenzuola d'Arda (PC) Italy; ^3^ The James Hutton Institute Dundee UK; ^4^ Department of Field Crops, Faculty of Agriculture University of Çukurova Adana Turkey; ^5^ Global Crop Diversity Trust Bonn Germany; ^6^ Department of Earth and Environmental Sciences, Manchester Institute of Biotechnology University of Manchester Manchester UK

**Keywords:** ancient DNA, domestication bottleneck, genomics, *Hordeum*, phylogeography

## Abstract

Barley is one of the founder crops of the Neolithic transition in West Asia. While recent advances in genomics have provided a rather detailed picture of barley domestication, there are contradictory views on how the domestication process affected genetic diversity. We set out to revisit this question by integrating public DNA sequencing data from ancient barley and wide collections of extant wild and domesticated accessions. Using two previously overlooked approaches – analyses of chloroplast genomes and genome‐wide proportions of private variants – we found that the barley cultivated six millennia ago was genetically unique and more diverse when compared to extant landraces and cultivars. Moreover, the chloroplast genomes revealed a link between the ancient barley, an obscure wild genotype from north‐eastern Libya, and a distinct population of barley cultivated in Ethiopia/Eritrea. Based on these results, we hypothesize past existence of a wider North African population that included both wild and cultivated types and suffered from genetic erosion in the past six millennia, likely due to a rapid desertification that ended the Holocene African humid period. Besides providing clues about the origin of Ethiopian landraces, the hypothesis explains the post‐domestication loss of diversity observed in barley. Analyses of additional samples will be necessary to resolve the history of African barley and its contribution to the extant cultivated gene pool.

## Graphical abstract

Barley cultivated 6 millennia ago was more diverse than extant cultivars and landraces. Private variants and chloroplast haplotypes indicate strong genetic erosion in North Africa and link together ancient barley, extant Ethiopian landraces and a rudimentary wild population of Cyrenaica.

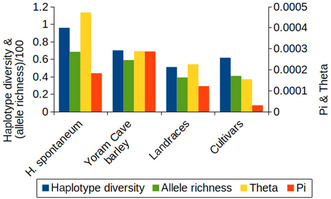

## 1 INTRODUCTION

Barley (*Hordeum vulgare* L.) is the fourth most cultivated cereal crop worldwide (FAOSTAT, 2022), mainly used for animal fodder and beer brewing. It is also one of the first crops domesticated about 10,000 years ago in the Fertile Crescent alongside the related staple crops einkorn, emmer and bread wheat (Zohary et al., 2012), making it an important model for studying the domestication of cereals. Understanding the population genetic processes that accompanied crop domestication is essential for our appreciation of the extant crop diversity and guides the conservation and efficient utilization of this diversity. The traditional view of domestication recognizes that the genetic diversity of a crop is smaller in comparison to its progenitor, and ascribes this reduction to the ‘genetic bottleneck’ of domestication (Meyer & Purugganan, 2013). The cause of the bottleneck is twofold – subsampling (only a fraction of the entire wild progenitor species takes part in the domestication) and selection (conscious cherry‐picking of individuals with the desired traits or unconscious selection of germplasm best suited for local environments and cultivation practices).

Conventionally, the severity of the diversity loss has been evaluated by comparing the diversity of modern crop varieties against extant lineages of the wild progenitor species. For cultivated barley and its wild progenitor *H. spontaneum*, early assessments of the domestication bottleneck varied from severe (Kilian et al., 2006) to modest (Morrell et al., 2014), with large uncertainties due to limited sampling of germplasm and genetic loci. Genome‐wide SNP and exome analyses on much larger sample sets concluded that barley landraces retained 73% and 80% of the wild barley diversity (Russell et al., 2011, 2016, respectively). However, a different picture is offered when a diversity survey is not limited to gene regions. A recent genotyping‐by‐sequencing analysis of virtually the entire German federal *ex situ* barley collection at IPK Gatersleben found 127,408 single nucleotide polymorphisms (SNPs) in 1140 wild barleys and 76,102 SNPs in 19,778 domesticated barleys (Milner et al., 2021). This translates to an average of ~4 and ~111 SNPs per cultivated and wild accession, respectively, indicating that domestication was a very narrow funnel for genetic diversity.

Recent analyses of ancient DNA (aDNA) from archaeobotanical samples raised questions about the diversity loss observed in modern crops, whether this is attributable to a domestication bottleneck and whether such a period of severe diversity reduction indeed accompanies early stages of domestication (Allaby et al., 2019; Brown, 2019; Smith et al., 2019). While archaeological plant remains with a level of preservation that allows DNA analysis are extremely rare, a recent excavation at Yoram Cave in the Judean Desert (Israel) found a single undisturbed anthropogenic layer of Chalcolithic origin (ca. 6200–5800 cal BP) containing a rich assemblage of more than 100 well‐preserved plant species, including barley (Mascher et al., 2016). Genomic analysis of five barley seeds concluded that the ancient genotypes differ little from landraces grown in present‐day Israel, and there has been no major change in the genetic composition of cultivated barley over the past six millennia (Mascher et al., 2016). Although this finding does not address the intensity of the domestication bottleneck, it suggests there has been no additional diversity loss since domestication. Subsequently, Allaby et al. (2019) examined the Yoram Cave barley data further using per‐individual proportions of heterozygous sites as an estimate of genetic diversity. Their comparisons of ancient, extant wild and cultivated barley samples led them to question the existence of a rapid and strong domestication bottleneck, and conclude that the loss of diversity occurred gradually and continuously after domestication.

The possibility that past environmental factors could be responsible for changes in the distribution range and diversity of a crop and its wild progenitor is usually sidelined due to lack of direct evidence. However, it has been established that regions adjacent to the Fertile Crescent went through substantial environmental changes during and after the period of crop domestication. Throughout much of the Holocene, large areas of North Africa and the Arabian Peninsula consisted of grassland/steppe and wooded grassland/savanna (Amrani, 2018; Hoelzmann et al., 1998; Pausata et al., 2020; Watrin et al., 2009). This ‘African humid period’ ended abruptly in the sixth millennium BP, due to orbitally forced decline in monsoon strength (Hildebrand et al., 2018; Hoelzmann et al., 1998; Pausata et al., 2020). The subsequent desertification of the region could have reduced the natural distribution range of wild barley and shifted the cultivation zones of domesticated barley. An assessment of genetic erosion in the Western Asian crops should therefore consider possible habitat loss due to environmental change.

Here, we explore the question of diversity erosion in barley, using previously overlooked approaches and taking advantage of the Yoram Cave aDNA data. First, we reconstruct four virtually complete and one partial chloroplast genome of the Yoram Cave samples and integrate their haplotypes within a collection of almost 800 wild and cultivated accessions. Secondly, we use two largely non‐overlapping genome‐wide datasets of barley diversity to assess the distribution of private variants (PVs, i.e. variants carried by a single individual within sampled genomes) in both ancient and extant barley samples, and use the proportion of PVs as a measure of samples' uniqueness. These analyses provide multiple estimates of genetic diversity in a geographic context and allow inferences about population history. Signs of genetic erosion in North Africa led us to speculate about the natural distribution range of wild barley throughout the Holocene, the origin of cryptic diversity in North Africa, and the possible impact of Saharan desertification on the diversity of wild and cultivated barley.

## 2 MATERIALS AND METHODS

### 2.1 Chloroplast genome reconstruction

For reconstruction of the chloroplast genomes, we compiled 804 Illumina sequencing datasets. The majority of these consist of exome capture sequencing libraries, where complete chloroplast genome reconstruction is possible from the unspecific (off‐target) fraction of the reads, thanks to the multicopy nature of organellar DNA. We downloaded 397 exome datasets published by Bustos‐Korts et al. (2019) and Bretani et al. (2020) (NCBI BioProject PRJEB33527), 251 exome datasets published by Russell et al. (2016) (NCBI BioProject PRJEB8044), 15 exome datasets published by Mascher et al. (2013) (NCBI BioProject PRJEB1810), 46 exome datasets previously produced by Terry Brown's lab (Civáň et al., 2021; NCBI BioProject PRJNA389721) and 89 additional exome datasets prepared by Joanne Russell's lab (Chen et al., 2022; NCBI BioProject PRJEB53544). Only 12 samples are duplicated across these datasets. We also attempted to reconstruct chloroplast genomes from all ten radiocarbon‐dated Yoram Cave barley seeds described by Mascher et al. (2016; NCBI BioProject PRJEB12197). Five of these assemblies were successful: JK2282 (4225–4006 cal BC), JK3009 (3939–3775 cal BC), JK3010 (3940–3773 cal BC), JK3013 (4048–3976 cal BC) and JK3014 (3886–3708 cal BC). The chloroplast genome of a Tibetan landrace ‘Goumang’ was reconstructed from the whole‐genome sequencing data (Zeng et al., 2015; NCBI Study SRP055042). All public datasets were downloaded from the Sequence Read Archive (https://www.ncbi.nlm.nih.gov/sra) in the fastq format using sra toolkit 2.8 or later. Additional details about the biological samples are given in Table S1 (Supporting Information).

Our reference‐based chloroplast genome reconstruction followed a highly efficient pipeline that we have used previously for rice (Civan et al., 2019). First, duplicated (i.e. identical) read pairs were collapsed to a single copy with tally, using the ‐‐with‐quality flag (Davis et al., 2013), and subsequently quality‐trimmed by Trimmomatic (Bolger et al., 2014) in the paired‐end mode, using the ILLUMINACLIP function to remove adapter fragments. After trimming, the paired‐read datasets were converted into interleaved format using seq_crumbs except the (overlapping) ancient DNA read pairs that were merged into single reads with PEAR (Zhang et al., 2014). Subsequently, chloroplast genome‐matching reads were extracted in silico with the filter_by_blast script in seq_crumbs (using the cv. ‘Morex’ chloroplast genome EF115541 as a custom database, reverse filtering option and E‐value limit of 1e‐8). The chloroplast genome‐matching reads were imported into Geneious 6.1 (Biomatters; http://geneious.com) and mapped to the chloroplast genome reference (EF115541; the second inverted repeat removed), using 15 nt word length, allowing 2% gaps and mismatches (each) per read, mapping paired reads only (except for the merged aDNA datasets) and using two assembly iterations. The chloroplast genome was called separately for each dataset as the 75% consensus of the assembly. The consensus sequences were aligned together with the public *H. jubatum* chloroplast sequence (NC_027476) used as an outgroup. The alignment of 805 chloroplast genomes (804 assemblies and the outgroup) was visually checked along its entire length of 121,495 bp, and misaligned regions were corrected manually. We found that a ~6.3 kb region of the chloroplast genome has a diverged paralogous copy in the mitochondrial genome, reads from which interfere with the chloroplast DNA assembly. This region, spanning the genes *atpA*, *rps14*, *psaB* and *psaA*, was therefore excluded from the downstream analysis. Additionally, intergenic regions (which frequently contain microsatellites and homopolymers) were removed together with RNA genes (that jointly contain only two variable positions with very low minor allele frequencies), leaving only protein‐coding genes (including introns) with a total length 57,868 bp for further analysis. These data were used to calculate nucleotide diversity (π; *Pi* and Watterson's *Theta*) in variscan 2.0.3 (Hutter et al., 2006) separately for cultivars (213 accessions), landraces (411), Yoram Cave samples (5) and *H. spontaneum* (147), including sites with gaps and ambiguities, and the NumNuc parameter set to 2.

Additionally, we extracted polymorphic sites with the minor variant present in ≥2 samples (treating gaps as missing data) in order to construct a median‐joining (MJ) network (Bandelt et al., 1999) and calculate haplotype diversity using the formula

$$H=\frac{N}{N-1}\left(1-\sum_{i}x_{i}^{2}\right)$$

where *x*_*i*_ is the frequency of haplotype *i* and *N* is the sample size of the specified group. Allelic richness was calculated according to Foulley and Ollivier (2006), counting each haplotype as an allele. All diversity estimates include duplicated samples (see below) that in some cases yielded different chloroplast haplotypes, but exclude accessions suspected to be wild‐cultivated hybrids or feralized forms of cultivated barley. Those were recognized on the basis of a genome‐wide principal component analysis (PCA) (Patterson et al., 2006).

### 2.2 Distribution of private variants (nuclear genome)

Initially, we considered SNPs and indels carried by a single individual in a sample set, either in a heterozygous (singletons) or in a homozygous (private doubletons) state. However, based on the initial tests, we excluded the singletons and use the term ‘private variants’ for private doubleton SNPs and indels only. We examined the proportions of PVs within the total number of sites with valid (non‐missing) data points per sample. Since modern and aDNA samples differ substantially in their proportions of missing data, this calculation was preferred to the one used previously (Cubry et al., 2017), where each individual was characterized by its contribution of singletons to the entire dataset (population sample). We have used two VCF (Variant Call Format) files that differ in their proportions of traditional landraces, elite cultivars and wild accessions, and where PVs had not been removed by a minor allele frequency/count filter. The first is the ‘base dataset’ published by Civáň et al. (2021) focused on the diversity of barley landraces and *H. spontaneum*. It contains the Yoram Cave sample with the highest sequencing depth (JK3014) alongside 309 non‐redundant modern accessions mapped on Morex pseudomolecules v1.0 (Mascher et al., 2017). The second VCF file focused on the diversity of modern cultivars was prepared specifically for the purposes of this paper by integrating 397 barley exome datasets of the Whealbi project (Bretani et al., 2020) and the whole‐genome sequencing data from the five Yoram Cave seeds (JK2281, JK3009, JK3010, JK3013, JK3014). Briefly, exome read quality was assessed with FastQC (Andrews, 2010) and adapter sequences were removed with Trimmomatic v0.36 (Bolger et al., 2014), simultaneously trimming both ends to a base quality of 20. Processing of the aDNA datasets was identical to the pipeline used for the chloroplast genome reconstruction. Pre‐processed reads were mapped against the Morex v3 reference (Mascher et al., 2021) with bwa‐mem 0.7.17 (Li, 2013). Duplicated reads from the resulting BAM files were marked using the MarkDuplicates command of Picard. Variant calling and read realignment around indels were performed with GATK v.4.3.0.0 (https://www.broadinstitute.org/gatk/; McKenna et al., 2010), following best practices. Per‐sample variant calls limited to the exome capture target space were obtained with the HaplotypeCaller tool of GATK. The resulting per‐sample GVCF files were consolidated in a unique GATK database using the GenomicsDBImport tool. Finally, the joint genotyping of samples was carried out using the GenotypeGVCFs tool to produce a set of joint‐called variants further filtered with the following parameters: SOR > 3; MQ < 50; MQRankSum < −8; ReadPosRankSum < −5; DP > 29,300; QUAL <30. Subsequently, sites with >50% missing genotype calls were removed. Since barley is a predominantly self‐pollinating species (a mating system that leads to homozygotization), positions with a high frequency of heterozygous calls indicate read‐mapping problems rather than true heterozygosity. Positions with the proportion of heterozygous calls ≥5% with respect to all non‐missing calls were therefore also removed. These steps led to a VCF file referred to as the ‘whealbi dataset’ in this paper.

Prior to the analysis of the PVs, we identified and excluded duplicated samples, which can strongly bias individual estimates (duplicated samples have no true PVs). First, we created an IBS (Identity By State) distance matrix using plink1.9 (Purcell et al., 2007), with the flat‐missing option. Within this matrix, we searched for IBS distance between known duplicates (based on passport data), which was >0.985 in both datasets. We therefore chose this value as a threshold for duplicate identification, and for each group of samples with IBS distances above this threshold, we retained only the sample with a lower proportion of missing genotypes. PVs were counted with vcftools v4.2 (Danecek et al., 2011), testing different depths for genotype calling (minDP 2–5).

## 3 RESULTS

### 3.1 Ancient barley in the context of the extant chloroplast diversity

**FIGURE 1:**
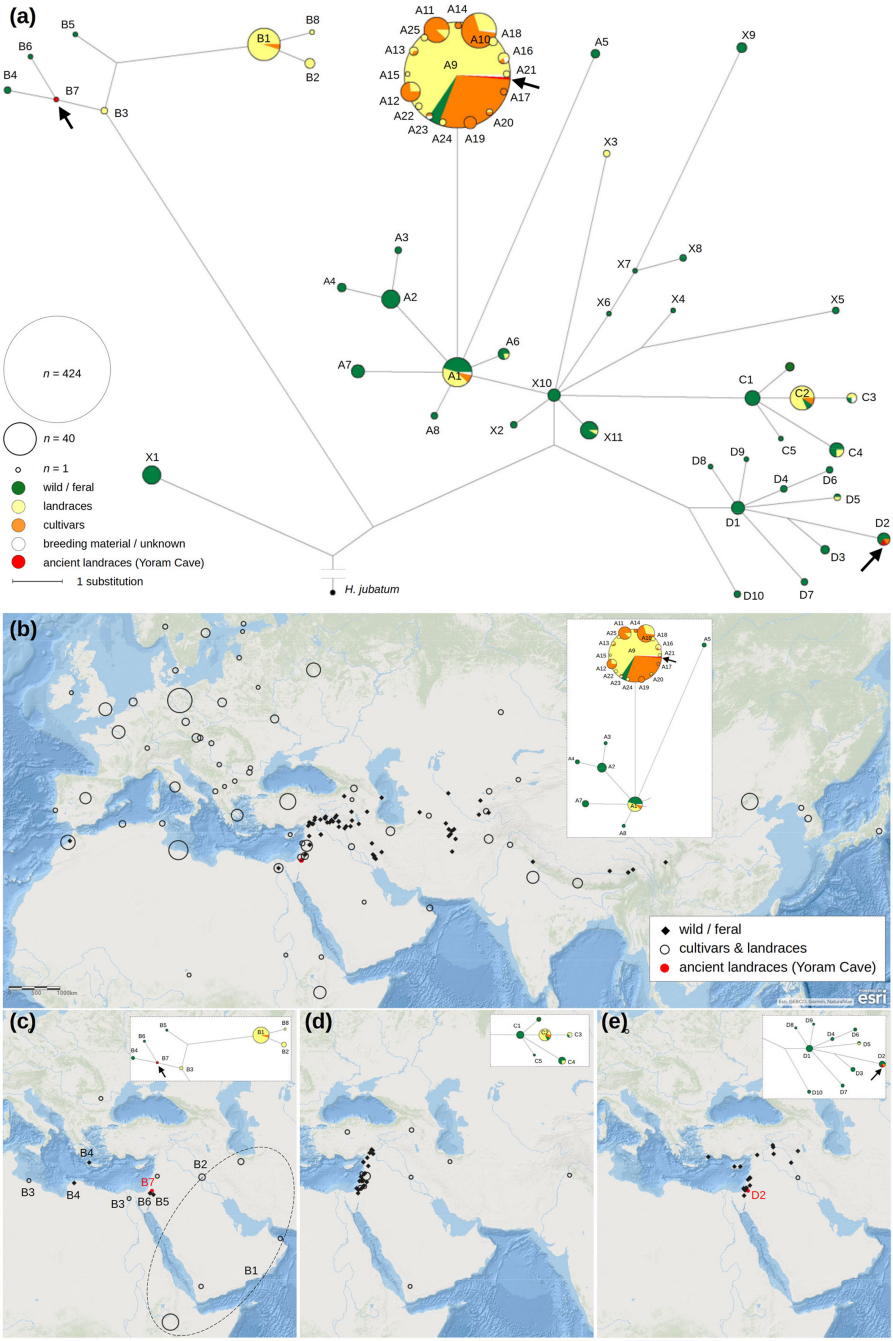
Genealogy of the barley chloroplast haplotypes and their geographic distribution. (a) Median‐joining network constructed from 116 variable positions in the chloroplast dataset. Edge lengths are proportional to the number of substitutions; node sizes are proportional to the number of samples. Haplotype codes are adjacent to the haplotype nodes, with samples listed in Table S1 (Supporting Information). Black arrows indicate the positions of the five Yoram Cave haplotypes. (b) Geographic distribution of the A‐haplogroup, showing domesticated barley as size‐proportional circles centred on countries' capitals and wild accessions as individual data points. (c) Geographic distribution of the B‐haplogroup, as above. Most data points are marked with haplotype codes to aid phylogeographic inference. The occurrence of the B1 haplotype is indicated with the dashed ellipse. (d) Geographic distribution of the C‐haplogroup. (e) Geographic distribution of the D‐haplogroup.

We report here a reference‐based reconstruction of 804 barley chloroplast genome sequences (incl. duplicates), mostly from the unspecific fraction of exome‐sequencing data. Within an alignment of protein‐coding genes (exons and introns), we found 116 reliable segregating sites that yielded a well‐structured haplotype network (Figure 1a). We designated four haplogroups (A–D) that contain extant or ancient cultivated barley, and we distinguish individual haplotypes with appended numbers (e.g. A9). All haplotypes, including those not forming a formal haplogroup (labelled as ‘X[1–11]’), are listed in Table S1 (Supporting Information). The vast majority of cultivated barley accessions (83%; 524/632 non‐redundant extant cultivars and landraces) carry the A9 haplotype or one that is closely related (1 substitution). The geographic origins of the A‐haplogroup are unclear, as it has a very wide distribution in wild barley (Figure 1a,b). A group of landraces (39) mostly originating from Ethiopia, but also from the Arabian Peninsula, Iraq and Iran carry a very distinct haplogroup B (Figure 1a,c), separated from the A‐haplogroup by ≥15 substitutions. The entire B‐haplogroup stems from the haplotype B3 that was found in two accessions from Libya and Egypt with unclear domestication status (Russell et al., 2016). Related wild accessions were sampled from Libya, Jordan, Israel and the island of Crete. The third haplogroup C contains a substantial number of Middle‐Eastern landraces (22), which according to the distribution of wild accessions originated in the western arm of the Fertile Crescent (Figure 1a,d). Finally, haplogroup D comprised 25 wild accessions mainly from the Fertile Crescent plus two cultivated accessions that we discuss below (Figure 1e).

**FIGURE A1:**
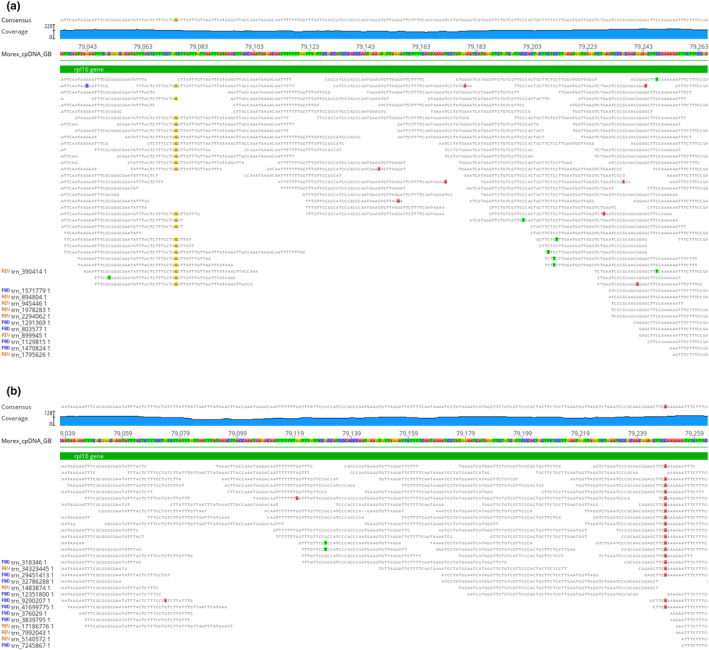
Quality of the ancient chloroplast genome reconstruction. (a) Excerpt of the reference‐based assembly showing a part of the *rpl16* gene for the Yoram Cave seed JK2282. (b) Excerpt of the reference‐based assembly showing a part of the same *rpl16* gene as above, but for the Yoram Cave seed JK3009. Read positions differing from the reference genome (Morex chloroplast genome EF115541; haplotype A9) are highlighted by coloured background. The depth of coverage and read quality allow genetic variants to be clearly distinguished from sequencing errors. The images were prepared in Geneious 6.1 (Biomatters; http://geneious.com).

We reconstructed chloroplast genomes for five Yoram Cave seeds (JK2282, JK3009, JK3010, JK3013, JK3014). Among the libraries that had been treated with uracil‐DNA glycosylase and deep‐sequenced (Mascher et al., 2016), high‐quality assemblies (containing no ambiguities at the 116 scored positions) were generated for seeds JK3009, JK3013 and JK3014. The seed JK3010 yielded a lower‐quality assembly (containing 31 ambiguities or missing data at the 116 positions), while seed JK2281 did not contain sufficient data for a reliable haplotype reconstruction. Among the libraries not treated with the glycosylase, seed JK2282 yielded a high‐quality chloroplast assembly (no ambiguities at the 116 positions; see Figure A1a) from only 18 million raw reads. The seeds JK3010, JK3013 and JK3014 were assigned to haplotype A9. However, JK2282 displays a unique haplotype B7 within the ‘Ethiopian’ B‐haplogroup, and JK3009 was typed as haplotype D2, which is absent in extant landraces but found in four wild barleys from southern Israel (Figure 1a,e). Each nucleotide variant in the JK2282 and JK3009 assemblies differing from the reference genome was checked visually, and unambiguous variant calling was confirmed in all cases (see assembly excerpts in Figure A1).

**FIGURE 2:**
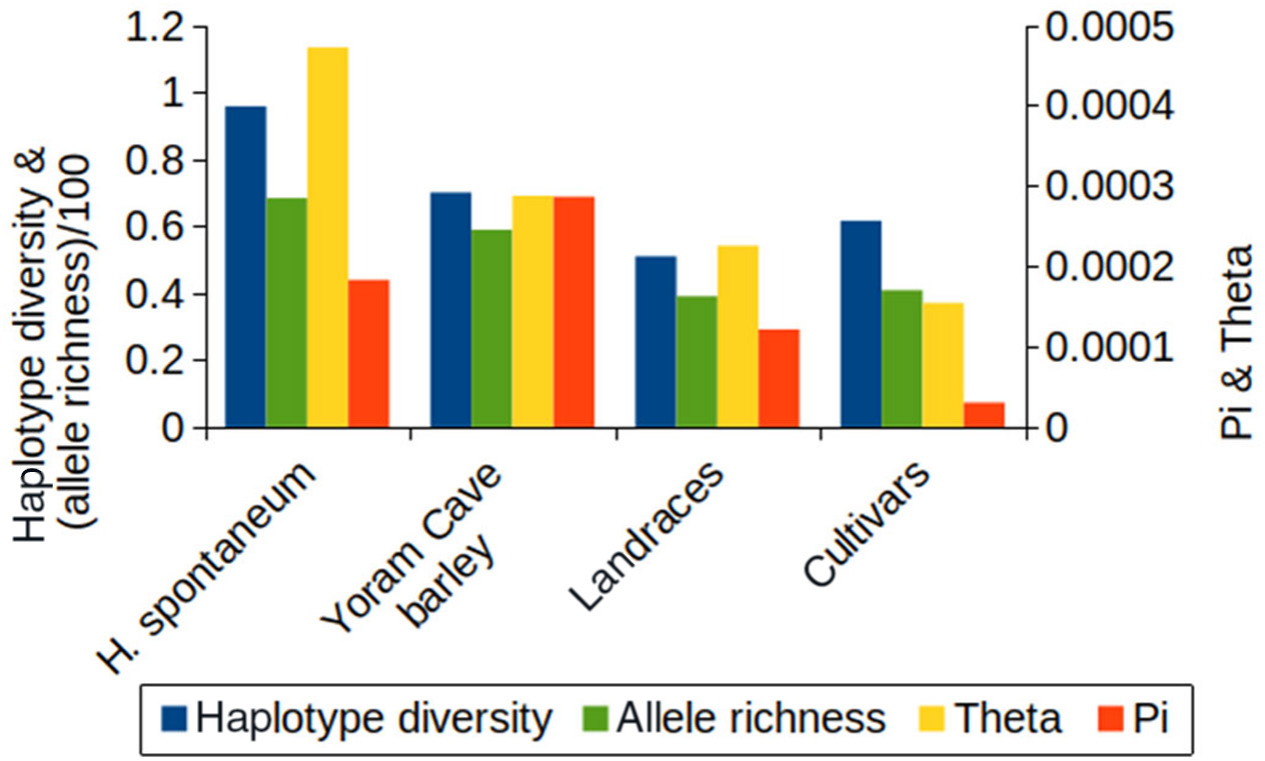
Measures of diversity for the barley chloroplast genomes.

Since the five ancient barley seeds from Yoram Cave carry three different chloroplast haplotypes from three genetically distant haplogroups, the diversity of the Yoram Cave barley is very high. All diversity measures of ancient barley are higher than the diversity of extant cultivars and landraces (Figure 2). While the Yoram Cave barley is the most diverse according to *Pi*, diversity measures that correct for sample size differences (allelic richness and *Theta)* place it second after wild barley.

### 3.2 Genome‐wide diversity

**FIGURE 3:**
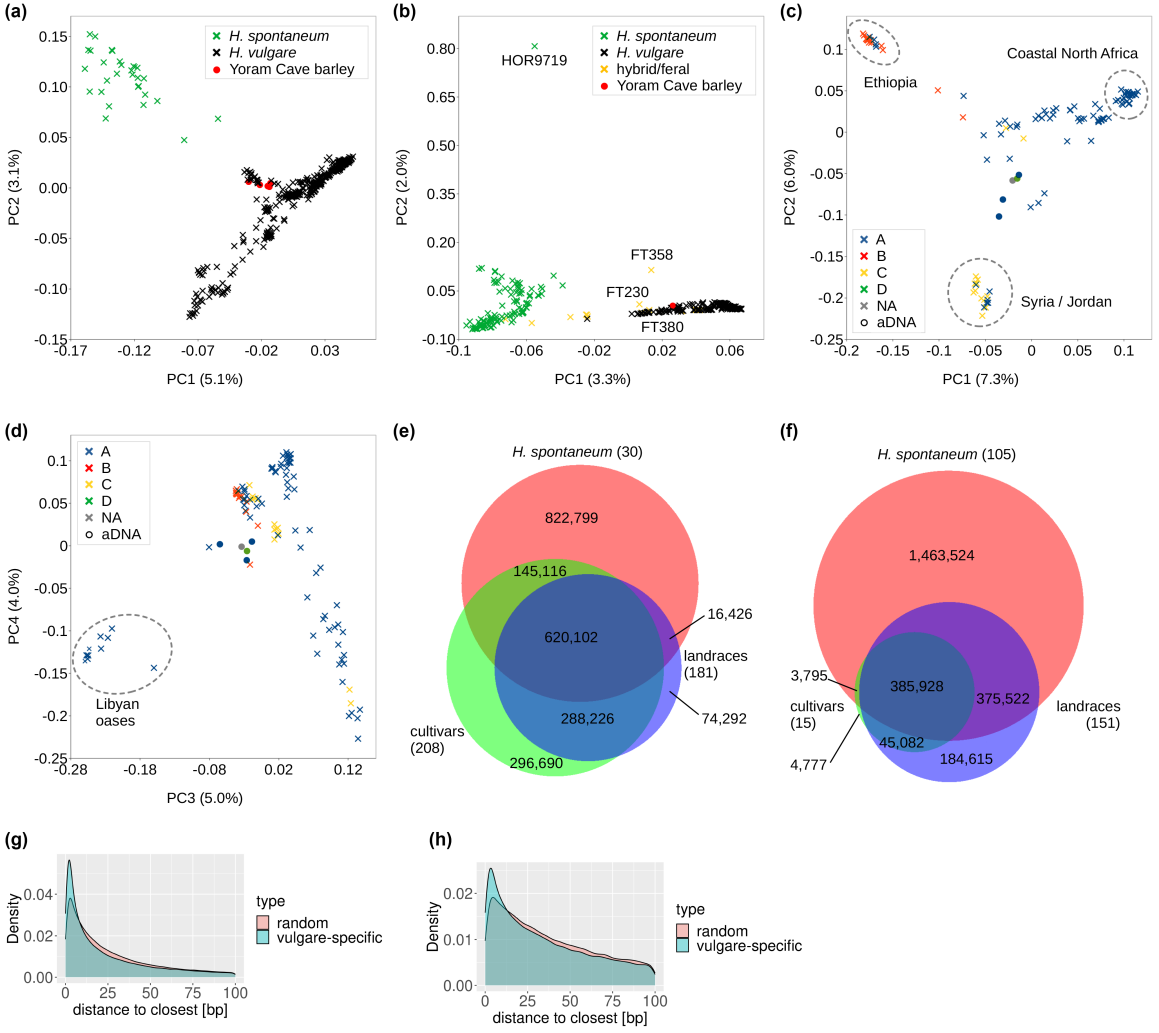
Distribution of genome‐wide diversity. (a) The top two principal components calculated from all samples (whealbi dataset) assign the Yoram Cave barley to the domesticated cluster. (b) The top two principal components calculated from the base dataset. The wild accession HOR9719 and suspected wild‐cultivated hybrids (in yellow) FT230, FT380 and FT358 are indicated, as these accessions are relevant for the North African diversity centre hypothesis (see Discussion). (c) The first and second PC calculated from the whealbi subset of cultivated samples separate the Ethiopian and Levantine landraces, roughly corresponding to the chloroplast haplogroups B and C, respectively. (d) The third PC from the same subsets further separates the barley collected at Libyan oases. Note that a chloroplast haplotype for the Yoram Cave seed JK2281 is not available, while the seed JK2282 with the B7 haplotype was not included in the whealbi VCF file due to insufficient data. (e) Venn diagram showing variable positions in cultivars, landraces and wild barley of the whealbi dataset, with SNP subsets and sample sizes indicated. (f) As above, but for the base dataset. (g) 659,208 *H. vulgare*‐specific SNPs (whealbi dataset) are physically closer to each other when compared to a randomly drawn SNP subset of the same size. (h) 234,474 *H. vulgare*‐specific SNPs (base dataset) are physically closer to each other when compared to a randomly drawn SNP subset of the same size.

Principal component analysis of the genome‐wide (nuclear) data clearly separated wild and domesticated barley and placed the Yoram Cave samples within the domesticated cluster (Figure 3a,b). Since the exome‐based population structure of extant barleys corresponds well to published results (Civáň et al., 2021; Pankin et al., 2018; Russell et al., 2016), here we restrict our attention to the positioning of the Yoram Cave barley with respect to cultivated accessions sampled from the Levant, Africa and the Arabian Peninsula. The top three principal components that jointly explain 18.3% of the total variation in the whealbi dataset distinguished four clusters that partially reflect the chloroplast haplogroups (Figure 3c,d; Figure S1, Supporting Information). The first PC separated the Ethiopian barley with the characteristic B‐haplogroup from the rest (Figure 3c). The second PC distinguished a particular population of 2‐rowed landraces sampled in Syria and Jordan, associated with the C‐haplogroup. The third PC separates barley landraces sampled at Libyan oases (central Libya) from those sampled along the Mediterranean coast (Figure 3d), all of which carry the A‐haplogroup. The barleys sampled in Morocco, Tunisia, Algeria, Egypt and coastal Libya are genetically similar to the Levantine A‐haplogroup barleys, which were apparently the source population for the expansion route along the North African coast. The Yoram Cave barley, having the three distinct chloroplast haplotypes, takes a rather central position with respect to the four PCA clusters. This could be due to its ancestral, pre‐divergence status, but it could also reflect the high proportion of missing data in four of the Yoram Cave seeds.

What portion of the wild diversity was carried over to *H. vulgare* during domestication and as a result of subsequent gene flow? This question can be addressed by a Venn diagram showing the sharing of variable sites by wild and cultivated barley; however, the results depend heavily on the intensity of *H. spontaneum* sampling (Figure 3e,f). In the whealbi dataset (30 *H. spontaneum*, 389 *H. vulgare*), close to 50% of the wild diversity is found in domesticated barley. When the sampling of wild barley is increased (105 *H. spontaneum*, 166 *H. vulgare*; base dataset), only 34% of the wild diversity is found in domesticated barley. This number drops to 24% when the sampling of wild barley is increased even further (266 *H. spontaneum*, 555 *H. vulgare*; dataset published by Chen et al., 2022; not shown). Thanks to a large number of *H. vulgare*‐specific variants (659,208 and 234,474 SNPs in the whealbi and base datasets, respectively), domesticated barley still appears relatively diverse. However, not all *H. vulgare*‐specific variants are true post‐domestication variants. Many of them could be variants that originated in wild barley but were not sampled here. This is indicated by the fact that many of the *H. vulgare*‐specific variants are tightly linked (Figure 3f,g), which is not expected for true post‐domestication SNPs. Tightly linked *H. vulgare*‐specific variants could emerge due to domestication founder effects or undersampling of wild haplotypes. These observations suggest that the domestication bottleneck in *H. vulgare* was rather severe (although the pace and timing of the diversity loss remain unclear), and a part of the genetic ancestry of *H. vulgare* is not represented by *H. spontaneum* accessions sampled here.

### 3.3 Genome‐wide proportions of PVs provide a measure of the uniqueness of individual samples

**FIGURE 4:**
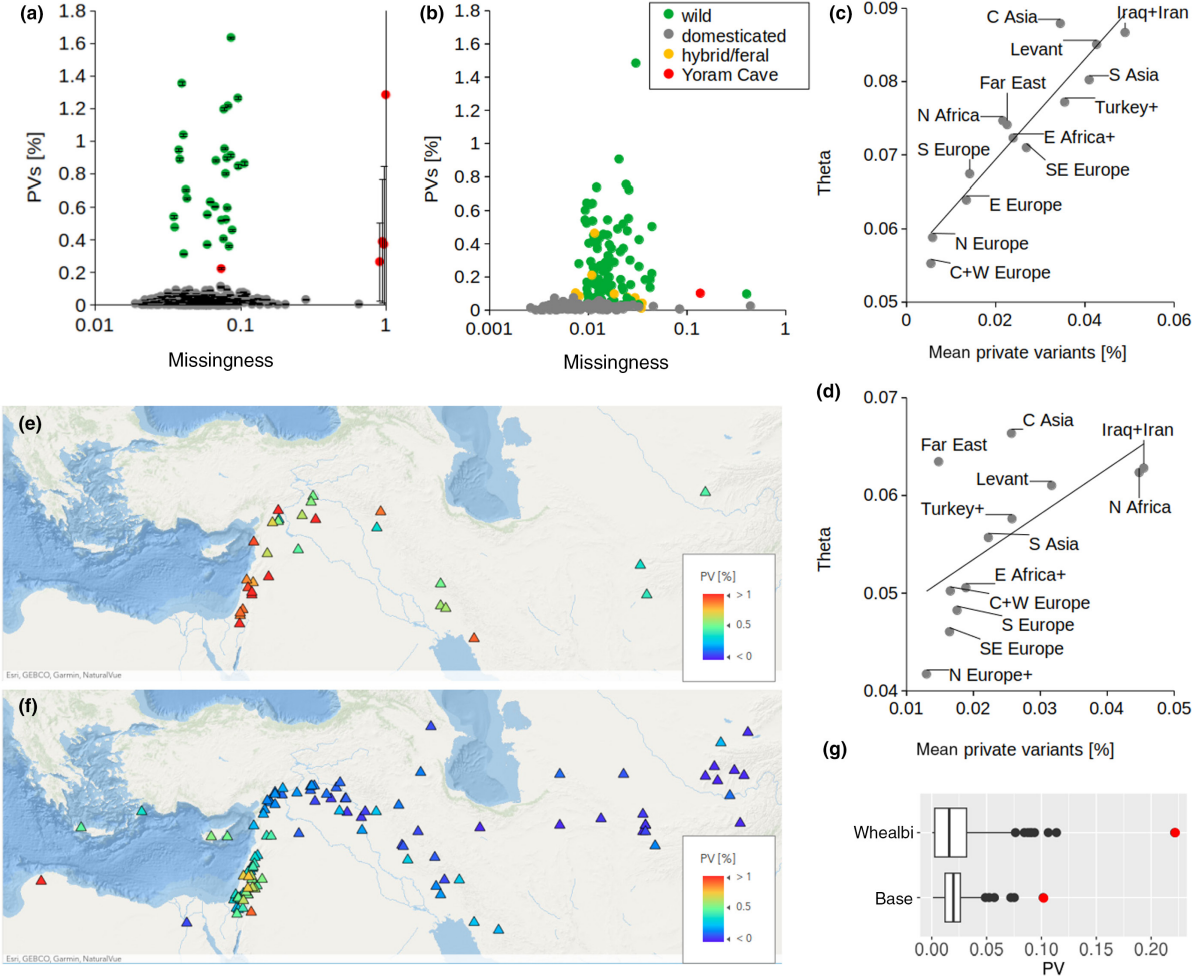
Uniqueness of barley samples measured as the proportion of PVs in the total number of non‐missing variant calls. (a) The lack of relationship between the proportions of PVs and missing genotype calls in the whealbi dataset. The scatter plot shows average values calculated at minDP2‐5, with error bars indicating standard deviation. (b) The lack of relationship between the proportions of PVs and missing genotype calls in the base dataset. (c) The relationship between the proportion of PVs and nucleotide diversity in the domesticated barleys of the whealbi dataset, averaged for geographic regions. ‘C Asia’ – Central Asia; ‘S Asia’ – South Asia; ‘Turkey+’ – Turkey and Transcaucasia; ‘N Africa’ – North Africa; ‘E Africa+’ – East Africa and the Arabian Peninsula; ‘N Europe+’ – Northern Europe and Russia; ‘S Europe’ – Southern Europe; ‘SE Europe’ – Southeastern Europe; and ‘C + W Europe’ – Central and Western Europe. (d) The relationship between the proportion of PVs and nucleotide diversity in the domesticated barleys of the base dataset, with geographic regions as above. (e) The proportions of PVs in wild barley of the whealbi dataset. (f) The proportions of PVs in wild barley of the base dataset. (g) Boxplots showing the distribution of the PV proportions within cultivated barley of the whealbi and base datasets. Outliers (Q3 + 1.5*interquartile range) are indicated as individual points, with JK3014 highlighted in red.

**FIGURE A2:**
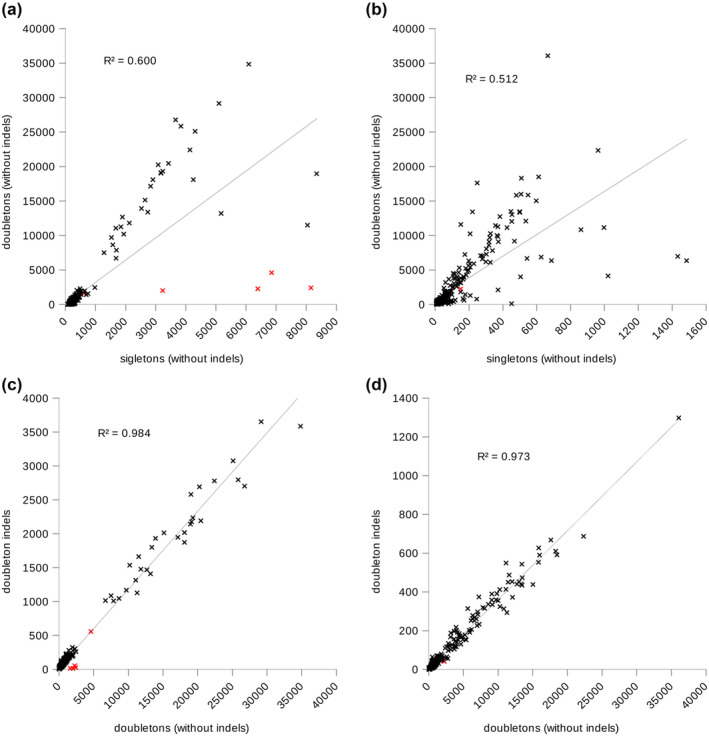
Comparison of different classes of PVs. Private doubletons (i.e. SNPs present in a single individual in homozygous state) are considered here as a reference for other classes. Black crosses represent extant wild and cultivated barleys; red crosses represent the Yoram Cave samples. (a) Comparison of singleton SNPs to doubleton SNPs across samples of the whealbi dataset. (b) Comparison of singleton SNPs to doubleton SNPs across samples of the base dataset. (c) Comparison of doubleton indels to doubleton SNPs across samples of the whealbi dataset. (d) Comparison of doubleton indels to doubleton SNPs across samples of the base dataset.

PVs are commonly excluded from population genetic datasets (by applying minor allele frequency/count filters) in part because they are considered unreliable and uninformative (e.g. in phylogenetics, PVs do not help to resolve tree topologies, and they merely extend the terminal branches). However, we expect PVs to reflect the genetic uniqueness of an individual within a particular population sample. To examine the robustness of PVs with respect to sequencing depth and variant calling thresholds, we performed several tests. We found that per‐sample counts of singletons (PVs in the heterozygous state) correlate poorly with the doubleton counts (homozygous PVs) (Figure A2a,b). Since heterozygous genotype calls in predominantly self‐pollinating species, such as barley, are likely to originate from somatic mutations, assembly and sequencing errors, we excluded singletons from the subsequent analyses. On the other hand, we found that homozygous private indels correspond well with the doubletons (Figure A2c,d), and so we grouped them together under the term ‘private variants’ used throughout this text. We found that for samples with <50% missing data (i.e. all modern samples and one ancient Yoram Cave seed), PV proportions are robust in relation to the minimum depth threshold for genotype calls (minDP 2–5) and do not correlate with the proportion of missing data even at minDP 2 (*r* = .065; *p* = .18; Figure 4a,b). However, for samples with extreme proportions of missing data (the remaining four Yoram Cave seeds with <1× sequencing depths), the statistic is very sensitive to the minDP filter and practically unreliable (Figure 4a).

In both datasets, the statistic is on average significantly higher in wild barley compared to domesticated barley (Figure 4a,b). Within domesticated barley, landraces have significantly higher PV proportions compared to cultivars (*t*‐test; *p* < 10^−5^). We conclude that the per‐sample proportion of PVs (excluding singletons) is a reliable measure of the genetic uniqueness of an individual within a given population sample, insensitive to the genotype depth threshold up to moderate levels of per‐individual missingness (<50%). Across geographic regions of barley cultivation, the proportion of PVs is significantly correlated with genetic diversity (*Theta*) in the whealbi and base datasets (*r* = 0.934, *p* < e10^−5^; *r* = .644, *p* < .05, respectively; Figure 4c,d). A scatter plot of Theta and the PV proportions can indicate regions with under‐/oversampled diversity, since disproportionately high or low proportions of PVs indicate very little or too much sampling redundancy, respectively. For example, domesticated barley sampled from Central Asia and the Far East has high levels of genetic diversity in both datasets, but relatively low proportions of PVs in the base dataset, suggesting these regions were oversampled. The proportion of PVs as a measure of sample uniqueness provides interesting information for wild barley, too. Both datasets indicate that the southern Levant harbours the highest diversity of wild barley. However, wild barley sampled from Libya and Mediterranean islands (Cyprus, Crete, Rhodes) shows relatively high levels of uniqueness, with the Libyan barley HOR9719 having the highest proportion of PVs (1.48%) in the base dataset. On the other hand, the eastern arm of the Fertile Crescent, together with Central Asia, appears oversampled in the base dataset.

**FIGURE A3:**
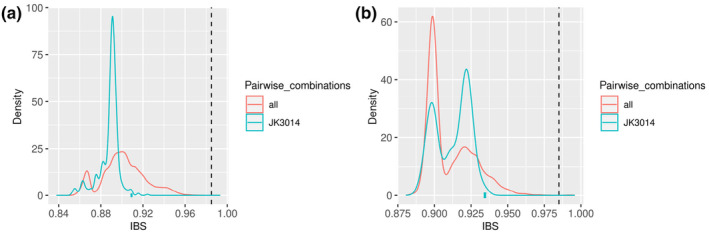
Comparison of pairwise IBS distances in the entire whealbi (a) and base (b) datasets (red line) to the IBS distances calculated between JK3014 and all other accessions in the respective datasets (turquoise line). High IBS values indicate high similarity; the vertical dashed line shows the threshold for duplicate removal (0.985). Both datasets are characterized by a bi‐modal distribution of IBS distances, with the first peak corresponding to wild‐cultivated accession pairs and the second corresponding mostly to cultivated‐cultivated pairs. In both datasets, JK3014 density plot is shifted to the left with respect to the entire dataset, indicating a lack of highly similar accessions. IBS distances between JK3014 and the three most similar accession (WB‐109, WB‐069 and WB‐333 for whealbi; PI356226, FT549 and BCC107 for base) are indicated with turquoise bars on the *x*‐axis.

Due to the extreme proportions of missing data in four of the five Yoram Cave seeds, only the sample JK3014 can be evaluated (Figure 4a,b). The proportion of PVs in JK3014 is higher compared to extant domesticated barley (*z*‐score 9.53 and 6.32 in the whealbi and base datasets, respectively), though not as high as in wild barley (*z*‐score − 1.71 and −0.72 in the whealbi and base datasets, respectively; suspected hybrid/feral accessions regarded as neither wild nor domesticated, see Table S1). Within cultivated barleys, JK3014 is an outlier with respect to the PV proportion in both datasets (Figure 4g), confirmed by the modified Thompson Tau test. This shows that the Yoram Cave barley is the most unique individual within the sampled extant domesticated barley. Assuming that the two datasets provide a good representation of extant diversity, the high proportion of PVs in JK3014 indicates erosion of genetic diversity in the past 6000 years. Based on an Identity‐By‐State matrix (a measure of genetic similarity) calculated from all sites in the filtered whealbi dataset (genotyped at minDP 3; incl. PVs), JK3014 is most closely related to the other Yoram Cave seeds (IBS 0.923–0.942). Compared to extant accessions, JK3014 is most similar to the landraces WB‐109, WB‐069 and WB‐333 (IBS 0.909‐0.91; Figure A3a) sampled from Syria, Morocco and Jordan, respectively. In the base dataset, JK3014 is most similar to the landraces PI356226, FT549 and BCC107 (IBS 0.934‐0.935; Figure A3b) sampled from Morocco, Jordan and Lebanon, respectively.

## 4 DISCUSSION

### 4.1 The domestication bottleneck

Domesticated barley is a relatively diverse crop (Civáň et al., 2021; Morrell et al., 2014), probably because of its wide distribution across varied climatic zones and its dual use as animal fodder and fermentable grain for beverage production. Population genetic analyses of genome‐wide diversity patterns have indicated that multiple populations of wild barley contributed to the genetic make‐up of this crop (Civáň et al., 2021; Pankin et al., 2018; Poets et al., 2015). Moreover, the tough rachis phenotype considered to be the crucial domestication trait in barley was achieved by at least three independent mutations, each producing the same effect (Civáň & Brown, 2017; Pourkheirandish et al., 2015). These observations suggest that the genetic base at the dawn of barley domestication was relatively wide, although still considerably narrower than the diversity of wild barley (Kilian et al., 2006; Milner et al., 2021; Morrell et al., 2014; Russell et al., 2011, 2016). It has been argued that selection, the second component of the domestication bottleneck besides subsampling, did not lead to a sudden decline in genetic diversity in the early phases of domestication (Allaby et al., 2019). Allaby et al. speculated that the diversity loss in annual crops was rather gradual and continuous and is better described as post‐domestication erosion. Is then the domestication bottleneck a myth (Brown, 2019)? Genome‐wide diversity data demonstrate that less than 50% of wild variants were retained by extant traditional landraces (Figure 3e,f). The diversity reduction from wild to domesticated barley is therefore obvious. However, only aDNA analyses can answer questions about the timing and pace of this reduction.

### 4.2 The Yoram Cave barley testifies to lost diversity

Mascher et al. (2016) produced genomic sequences from several 6000‐year‐old barley seeds that had been desiccated in the Yoram Cave (Israel). Ancient DNA data such as these are expected to provide direct evidence of past genomic, population and diversity changes. Mascher et al. (2016) concluded that the Yoram Cave barley is not substantially different from the lineages grown in present‐day Israel, which is consistent with the presence of a domestication bottleneck >6000 years ago, followed by limited change during the subsequent millennia of cultivation. On the other hand, Allaby et al. (2019) conclude that there is barely any discernible change in genetic diversity when the Yoram Cave barley is compared to extant wild and cultivated samples, suggesting absence of the domestication bottleneck altogether.

But what do we really know about the genetic identity and diversity of the Yoram Cave barley? Mascher et al. (2016) called Yoram Cave genotypes only at those positions that are variable in extant barley, perhaps assuming that variants not seen in high‐quality samples are unreliable. But if the Yoram Cave barley contains variants no longer present in extant barley, then those variants would have been missed. Moreover, assessing the diversity of a few aDNA samples, or more generally, the diversity of populations represented by only a few individuals, is inherently difficult because common measures of diversity (*Pi*, Watterson's *Theta*) are population‐, not individual‐based. An individual‐based measure of genetic diversity is therefore needed, particularly for scarce aDNA samples. In animals, an indication of population diversity and potential genetic erosion can be obtained by studying genome‐wide patterns of heterozygosity in an individual genome (Bosse & van Loon, 2022). Allaby et al. (2019) applied this approach to crop plants, using per‐individual proportions of heterozygous sites as a proxy of nucleotide diversity. However, such estimates of diversity are questionable in predominantly self‐pollinating crops, where homozygosity is the norm. In crops like barley, the proportion of heterozygous sites is generally very low, but can be dramatically increased by cross‐hybridization, with rapid decline in subsequent generations. Consequently, individuals from a highly diverse population can vary greatly in their proportions of heterozygous sites, depending on how deep in their genealogies cross‐pollination occurred. Moreover, this statistic is biased downwards in low depth aDNA samples, because heterozygous calls are likely to be missed at genotype depths <5.

One under‐exploited option for describing genetic diversity at the individual level is the frequency of PVs. Cubry et al. (2017) analysed the distribution of singletons in populations of African pearl millet. The study demonstrated that the proportion of singletons carried by an individual relative to all singletons in the population sample indicates that individual's contribution to the genetic diversity (Cubry et al., 2017). This individual‐based estimate can provide an overview of the diversity distribution across geographic space without the need to define populations. Since the proportion of rare derived variants is known to increase during population expansions (Keinan & Clark, 2012), singletons analysed in a Bayesian framework can ascertain the geographic origin of a range expansion (Cubry et al., 2017). However, singletons and PVs in general are a function of the individual's genotype and all other genotypes in the dataset. Therefore, PVs reflect the intensity of sampling and the genetic uniqueness of an individual within a sample set (a single representative of an exotic population will have many PVs while duplicated individuals will have none). Here, we applied this concept to assess the genetic uniqueness of ancient barley and to detect geographic regions with signs of undersampling and genetic erosion. We show that the Yoram Cave barley is unique in the context of extant barley diversity (the highest proportion of PVs among domesticated barley in both datasets). At least some of the PVs found in the Yoram Cave barley can be therefore regarded as variants that eroded from the domesticated gene pool during the last 6000 years.

Furthermore, we reconstructed five chloroplast genomes from the Yoram Cave sequence datasets. We show that high‐confidence assemblies are possible from aDNA samples even at low sequencing depths, thanks to the multicopy nature of plastid genomes. Although the chloroplast genome is considered to be a single locus (due to the absence of meiotic recombination), its length and relatively high diversity (especially in wild barley) allow haplotype identification, which can be quite informative in a phylogeographic context, analogous to the utility of mitochondrial DNA in the study of human migrations (Underhill & Kivisild, 2007). Comparisons with extant chloroplast genomes from cultivated barley revealed that the Yoram Cave barley has a higher haplotype and nucleotide diversity (Figure 2). Across Europe, 97.6% of sampled landraces and cultivars carry the haplotype A9 or one with a single substitution difference. In contrast, the five barley seeds collected from Yoram Cave – a single archaeological site spanning <500 years – contain three very distinct chloroplast haplotypes, two of which (B7 and D2) are virtually absent from the modern cultivated gene pool. The B haplogroup is discussed in detail in the section below. The D haplogroup is currently found only in the wild material, with the exception of an Iraqi accession (FT230; haplotype D5) that appears to be a wild×cultivated hybrid (Civáň et al., 2021), and a German cultivar (WB‐103; haplotype D2) that likely obtained its cytoplasmic genomes via breeding crosses. Interestingly, the D haplogroup is also found in wild barley from the Mediterranean islands Cyprus and Rhodes. This material is usually regarded as weedy forms that occupy secondary, disturbed habitats (Harlan & Zohary, 1966; Zohary et al., 2012). If this assumption is correct and wild barley found on the Mediterranean islands is a feralized form of imported domesticated barley, it further corroborates the assertion that the D haplogroup was once present in the cultivated gene pool.

**FIGURE A4:**
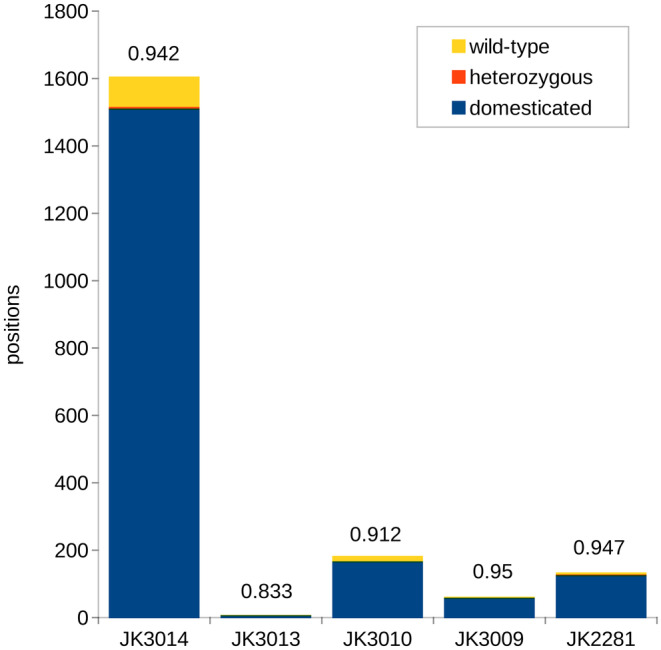
Proportions of wild type, domesticated and heterozygous variants in the Yoram Cave samples. Only polymorphisms with allelic frequency > 0.95 in domesticated barley and rare in wild barley (<0.25) were considered. If a Yoram Cave sample matches at a particular position the variant prevalent in domesticated barley, the position is considered domesticated. Otherwise, it is considered wild type. The proportions of domesticated alleles within all considered non‐missing alleles are indicated above the columns.

The analyses of PVs and chloroplast genomes therefore indicate that barley cultivated 6000 years ago was more diverse than our extant collections. It should be reiterated that the Yoram Cave barley appears to be fully domesticated. In addition to the PCA (Figure 3a,b), we found that JK3014 has a domestication variant in 94.2% of 1604 nuclear positions that are fixed across all domesticated barley (variant frequency > 0.95) but rare in wild barley (variant frequency < 0.25). A similar level of domestication was found in the other Yoram Cave seeds (Figure A4), including JK3009 which carries a very distinct chloroplast haplotype. While the strength of the genetic bottleneck caused by domestication remains unclear, the Yoram Cave data demonstrate that cultivated barley has lost some diversity since domestication, during the past six millennia.

Why that happened is unclear. In the case of the lost chloroplast haplotypes, adaptation‐driven selection seems to be an unlikely explanation. The JK2282 and JK3009 samples with the B7 and D2 haplotypes, respectively, differ from the reference haplotype A9 by only three non‐synonymous substitutions each. These substitutions are not likely to be selected against, since they have been retained in other B and D haplotypes. Additionally, chloroplast genomes are not genetically linked to nuclear loci; therefore, their genetic erosion cannot be due to the ‘sweeping’ effect of selection acting on other targets. Loss of neutral diversity due to genetic drift seems also unlikely, considering that over 2 million barley plants are usually sown and grown in a 1 ha field. Allaby et al. (2019) and Smith et al. (2019) propose that the gradual decline of diversity in crop species could be explained by a serial founder effect, a model originally proposed to explain the decrease in human genetic variation with increasing distance from East Africa (DeGiorgio et al., 2009; Ramachandran et al., 2005). However, this model also assumes small founding groups perturbed by genetic drift, which is difficult to imagine in the context of agricultural expansion (>2 million plants/ha). Besides, the observed geographic distribution of diversity does not clearly follow a serial founder effect. In some cases, domesticated barley from regions that are geographically distant from the Fertile Crescent is more diverse than barley from more proximal locations (e.g. Far East vs. Balkans; Central Asia vs. Turkey and Transcaucasia; see Figure 4c,d).

We argue that the diversity erosion in the past 6000 years of barley cultivation is neither the result of domestication‐related artificial selection nor caused by the genetic drift presumed to accompany agricultural spread. Instead, we speculate that the decline in diversity was caused by the desertification of Sahara and the Arabian Peninsula, which has occurred in the past 6000 years due to orbitally forced decline in monsoon strength (Hoelzmann et al., 1998; Pausata et al., 2020). Our phylogeographic analysis of the barley chloroplast genome diversity provides evidence supporting this hypothesis.

### 4.3 Signs of diversity erosion in North Africa

Traditional barley cultivation in present‐day Africa is concentrated in a few separate regions – including parts of Morocco, coastal areas of the Mediterranean Sea, Libyan oases, and the Horn of Africa (Ethiopia and Eritrea). Similarly, wild *H. spontaneum* has been reported from Libya (Maire, 1955), Morocco and Ethiopia (Helbaek, 1959; Zohary et al., 2012). The existence of a seemingly wild material in Morocco and Ethiopia alongside rather unique local landraces has stimulated hypotheses of independent barley domestication events in these regions (Molina‐Cano et al., 1999; Orabi et al., 2007). However, these claims have long been disputed (Badr et al., 2000), and modern sequencing methods have demonstrated that the wild barley collected in Morocco and Ethiopia is most likely feralized forms of the local landraces. Our base dataset contains some of the wild material from Molina‐Cano et al. (1999) (HS‐1, HS‐2, HS‐3 and HS‐8) and from Ethiopia (PI 356061), all of which clusters with cultivated barley instead of *H. spontaneum* (Civáň et al., 2021). We confirm that the seemingly wild material sampled from Morocco and Ethiopia is not autochthonous, but rather derived from cultivated barley.

However, the genetic identity of the Libyan wild barley is more complicated. Maire (1955) reported that *H. spontaneum* is common in the green mountains of Cyrenaica from Bardiyah and Tobruk to Benghazi, where it occupies clearings of forests, pastures and steppes on limestone hills and decalcified plateaus. This population is represented in the base dataset by the accession HOR9719 collected in 1981 and classified as *H. agriocrithon*, that is a six‐rowed wild barley. It has been shown that HOR9719 carries a domesticated variant of the *Btr2* gene (Civáň & Brown, 2017; Guo et al., 2022) and is considered to be a hybrid of wild and domesticated lines, similar to *agriocrithon* accessions from other regions (Guo et al., 2022). However, this Libyan accession HOR9719 is associated with wild barley along the first principal component and appears entirely unique along the 2nd (Figure 3b) and 3rd principal components. This accession does not stand out when PVs are removed by a minor allele frequency filter, highlighting the importance of PVs in detecting undersampled diversity. Accordingly, our results show that it has the highest uniqueness within the base dataset (1.48% of its genotype calls are PVs). Furthermore, it carries the chloroplast haplotype B4, which is absent in domesticated barley and found only in one additional wild accession from Crete. If it is indeed admixed, the hybridization must have involved a very exotic wild genotype that is not represented in our datasets. This genotype, now possibly extinct, was likely native to Libya.

The B haplogroup of barley chloroplasts offers an interesting case in phylogeography. It is a very ancient haplogroup that diverged from the reference haplotype A9 about 94,000 years ago, according to the nucleotide substitution rates and chloroplast genome fragment defined by Middleton et al. (2014). Most of the B‐haplogroup barleys are landraces collected from Ethiopia and the Arabian Peninsula, with some also from Iran and Iraq. The relationship of the Ethiopian barley with those from the Arabian Peninsula is well‐known and mirrors a similar relationship observed in landraces of emmer wheat (Badaeva et al., 2015). It has been proposed that the two crops spread to the Horn of Africa from Mesopotamia via the Arabian Peninsula (Badaeva et al., 2015).

Our network of chloroplast haplotypes reveals a different genealogy for the Ethiopian barley and the entire B haplogroup. All B‐haplotypes recovered in this study stem from the ancestral node B3. This node must have included wild barley in the past, but the B3 sequence is currently found only in two accessions with ambiguous domestication status from Egypt and Libya. As reported above, a closely related haplotype B7 was found in one of the Yoram Cave samples. The most frequent B‐haplotype (B1) is typically found in Ethiopian barley landraces, while two B2 landraces from Iraq appear to be recently derived from B1. Although the B‐haplogroup has obviously evolved in wild barley (according to the divergence estimate), it is presently found in only a few *H. spontaneum* accessions from Libya and Crete (B4), Jordan (B5) and southern Israel (B6), and is entirely absent from wild populations in Central Asia and the rest of the Fertile Crescent. We interpret these observations as follows: the B‐haplogroup evolved in wild barley in relative isolation from the Fertile Crescent populations, in a region from the Negev desert (Israel/Jordan) in the east to Cyrenaica (Libya) in the west. As evidenced by the Yoram Cave seed JK2282, the B‐haplogroup was present in the cultivated gene pool six millennia ago, probably thanks to post‐domestication gene flow between wild and cultivated barley. After the neolithic transition, the B‐haplogroup was possibly common in the region and gradually spread to Nubia and present‐day Ethiopia. The B‐haplogroup barleys found on the Arabian Peninsula and Iraq/Iran descended from the Ethiopian material, possibly in more recent times. The uniqueness of the Libyan wild barley, the absence of extant wild barley in the nodes B1 and B3, and the loss of the Yoram Cave haplotype B7 from the cultivated gene pool all suggest that the B‐haplogroup has suffered strong genetic erosion in the last 6000 years.

The hypothesis of a North African diversity centre for wild barley and its demise in recent millennia is consistent with the onset and termination of the Holocene African humid period. Throughout much of the Holocene, most of the area of present‐day Sahara, Sinai and the Arabian Peninsula consisted of grassland/steppe and wooded grassland/savanna (Hoelzmann et al., 1998; Pausata et al., 2020; Watrin et al., 2009), an ecosystem that could have been similar to the niches wild barley occupies today within the Fertile Crescent (Harlan & Zohary, 1966; Jakob et al., 2014). It is therefore possible that the present‐day Negev desert, Sinai peninsula, northern Egypt and Cyrenaica contained autochthonous populations of wild barley that had evolved in that region during the Late Pleistocene. If that was indeed the case, the gradual desertification of North Africa between 5400 and 4500 BP (Hildebrand et al., 2018; Pausata et al., 2020) would then have caused near‐extinction of the wild B‐haplogroup populations, whose past existence is documented only by a rudimentary population in Cyrenaica.

Interestingly, the B‐haplogroup barley escaped the desertification and survived to modern times also in the form of Ethiopian cultivated barley. How and when the domesticated B‐lineage was transferred to the Horn of Africa is not entirely clear. The near‐disappearance of the B‐haplogroup from Egypt/Libya – the region of the distal origin of Ethiopian barley – implies that the migration event preceded, or was triggered by the desertification of North Africa. The pattern of the desertification fits well with this proposition. West Asian crops (wheat and/or barley) start to appear in the archaeobotanical record of Lower Egypt around 7800 BP (Hassan, 1985). About 2000 years later, the Holocene humid period was drawing to an end. As the monsoon rains retreated south after 5200 BP, Egyptian Sahara became increasingly inhospitable (McDonald, 2016; Wetterstrom, 1998). But farther south in Sudan, in the path of the rain's retreat, conditions remained favourable. Some regions unoccupied until 6000 BP became inhabited, with main settlements appearing after 5000 BP (Wetterstrom, 1998), suggesting migration from more arid regions. Neolithic communities of this region are generally considered to be pastoralists who did not grow domesticated crops, although no sites have been sampled for plant remains (Wetterstrom, 1998). Microfossil evidence shows that wheat and/or barley were present in Northern Sudan since at least 7000 BP (Madella et al., 2014), but clear evidence of cultivation appears <6000 BP (Fuller & Lucas, 2021). However, as the aridity of the region increased, Sudanese settlements were also abandoned about 3000 BP (Wetterstrom, 1998). We surmise that with the progressive aridification of the region, the B‐haplogroup lineage of barley moved ever more south and eventually entered Ethiopia before 3500 BP (Arthur et al., 2019; Beldados et al., 2023), where it persisted in the form of local landraces to present times.

Our PCA identified yet another distinct African population of domesticated barley. It is formed by 6‐rowed barley sampled from oases scattered across central and southern Libya. This population has been recognized in a previous microsatellite‐based analysis (Pasam et al., 2014) and is genetically different from both the Ethiopian barley and the material sampled in the coastal regions of North Africa (Figure 3c, Figure S1, Supporting Information). The barley of the Libyan oases is also unrelated to the wild population of Cyrenaica, the palaeodistribution of which probably did not extend to the central Sahara, as it is absent in the archaeobotanical record of the early and middle Holocene of the region (Amrani, 2018; Fornaciari et al., 2018). We point out the possibility that the barleys from the Libyan oases are remnants of the crop introduced to the region by the Garamantian culture in the 1st millennium BC (Pelling, 2008). Based on archaeobotanical evidence, the barley of the Garamantes was hulled and 6‐rowed (matching the extant samples; see Pasam et al., 2014) and cultivated under a sophisticated arable regime relying on ‘foggara’ irrigation (Pelling, 2008; van der Veen, 1992). All our samples collected from the Libyan oases carry the common A9 haplotype, which is also present in all cultivated barley sampled from the coastal regions of North Africa. This indicates that the extant landraces of the Libyan oases descended from the material of the Levantine‐Mediterranean expansion route, an ancestry consistent with the observation that Garamantian agriculture was largely based on West Asian crops (Pelling, 2008; van der Veen, 1992). The clear genetic divergence of this population revealed by PCA can probably be ascribed to a strong selection pressure to adapt to the extreme Saharan environment and irrigation‐based farming.

### 4.4 Concluding remarks

Considerations of the past and present natural distribution range of *H. spontaneum* often overlook regions of North Africa despite reports of a seemingly autochthonous population in Cyrenaica that remains undersampled and poorly characterized to this day. Moreover, our knowledge about the history of barley cultivation in North Africa is limited due to the scarcity of archaeobotanical studies and considerable gaps in the data, both temporally and spatially (Pelling, 2016). In the context of the radically different environmental conditions of the region during the Holocene, this knowledge gap can be substantive and distort our understanding of barley origins, spread and the sources of its extant diversity. In this paper, we used ancient DNA samples to document diversity loss during the past six millennia of barley cultivation, and we revealed a connection between this lost diversity, an obscure wild barley from Cyrenaica, and the distinct Ethiopian population of cultivated barley of unknown origin. This link implies the existence of a North African population of wild barley that genetically contributed to the cultivated gene pool. Probably due to the rapid desertification that followed the Holocene African humid period, the African barley suffered genetic erosion and survives to this day in the form of an admixed wild population in Libya and traditional landraces of Ethiopia. Since these conclusions are largely based on the chloroplast DNA lineage and a limited number of samples (a single wild barley from Libya; five chloroplast genomes from the Yoram Cave), the hypothesis of a North African diversity centre, its role in barley domestication and its subsequent demise needs further investigation. Public seedbanks hold several genotypes of wild barley collected in Cyrenaica between 1981 and 1990. Genomic data from these accessions and additional ancient seeds can be combined in the future with existing datasets to resolve their genetic origins and improve our understanding of the domestication and extant diversity of cultivated barley.

## AUTHOR CONTRIBUTIONS

**Peter Civan:** Conceptualization (equal); formal analysis (lead); methodology (lead); visualization (lead); writing – original draft (lead); writing – review and editing (equal). **Agostino Fricano:** Conceptualization (equal); data curation (lead); methodology (supporting); writing – review and editing (equal). **Joanne Russell:** Conceptualization (equal); funding acquisition (equal); writing – review and editing (equal). **Caroline Pont:** Methodology (supporting); writing – review and editing (equal). **Hakan Ozkan:** Conceptualization (equal); writing – review and editing (equal). **Benjamin Kilian:** Conceptualization (equal); funding acquisition (equal); writing – review and editing (equal). **Terence A. Brown:** Conceptualization (equal); funding acquisition (equal); writing – review and editing (equal).

## CONFLICT OF INTEREST STATEMENT

The authors declare no competing interest.

## Supporting information


Figure S1.

Table S1.

## Data Availability

All raw sequence data used in this publication are publicly available and identifiable by NCBI BioProject codes mentioned in the Material and Methods section, and SRA accession numbers listed in Table S1 (Supporting Information). The base VCF file is available at Mendeley Data (https://doi.org/10.17632/BBV2MDXWCR.1). The whealbi VCF file and a multiple sequence alignment of the reconstructed chloroplast genomes are available at Mendeley Data (https://doi.org/10.17632/f6bjrszvdh.1).
