# Supplementary figures and images for: Genetic erosion in domesticated barley and a hypothesis of a North African centre of diversity
Source: Ecol Evol. 2024 Aug 7;14(8):e70068. doi: 10.1002/ece3.70068 (PMC11303984; doi:10.1002/ece3.70068)

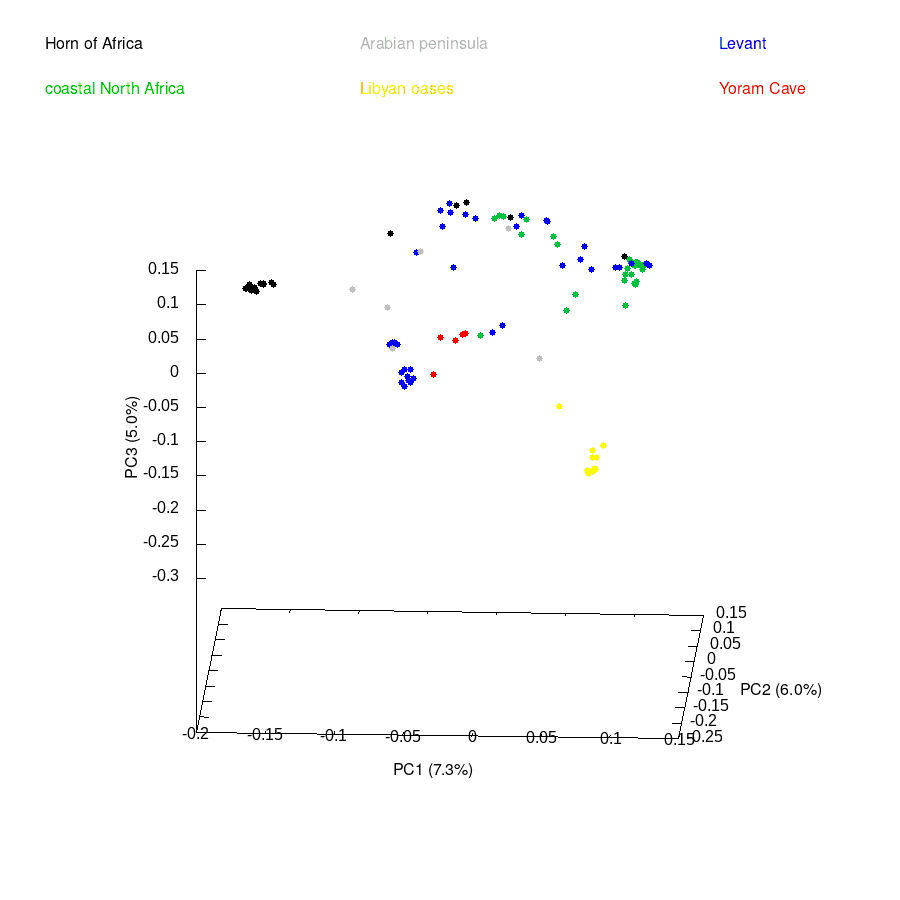

Supplement: Supplementary file 1 — Figure S1. [file ECE3-14-e70068-s002.gif]
